# Considering the societal perspective in economic evaluations: a systematic review in the case of depression

**DOI:** 10.1186/s13561-020-00288-7

**Published:** 2020-09-22

**Authors:** Juliane Andrea Duevel, Lena Hasemann, Luz María Peña-Longobardo, Beatriz Rodríguez-Sánchez, Isaac Aranda-Reneo, Juan Oliva-Moreno, Julio López-Bastida, Wolfgang Greiner

**Affiliations:** 1 AG 5 – Department of Health Economics and Health Care Management, Bielefeld University, School of Public Health, Universitaetsstrasse 25, 33615 Bielefeld, Germany; 2 Faculty of Law and Social Sciences, Economic Analysis Department, Research Group in Economics and Health, University of Castilla-La Mancha, Cobertizo San Pedro Mártir, S/N, 45002 Toledo, Spain; 3 Faculty of Technology and Science, University Camilo José Cela, Urb. Villafranca del Castillo, Calle Castillo de Alarcón, 49, 28692 Villanueva de la Cañada, Madrid, Spain; 4 Faculty of Social Science, Economic Analysis and Finance Department, Research Group in Economics and Health, University of Castilla-La Mancha, Avda. Real Fábrica s/n, Talavera de la Reina, 45600 Toledo, Spain; 5 Faculty of Health Science, Research Group in Economics and Health, University of Castilla-La Mancha, Av. Real Fábrica de Sedas, s/n, Talavera de la Reina, 45600 Toledo, Spain

**Keywords:** Cost-utility analysis, CUA, Quality-adjusted life years, QALY, Societal perspective, Incremental cost-utility ratio, ICUR, Direct costs, Indirect costs, Depression

## Abstract

**Background:**

Depressive disorders are associated with a high burden of disease. However, due to the burden posed by the disease on not only the sufferers, but also on their relatives, there is an ongoing debate about which costs to include and, hence, which perspective should be applied. Therefore, the aim of this paper was to examine whether the change between healthcare payer and societal perspective leads to different conclusions of cost-utility analyses in the case of depression.

**Methods:**

A systematic literature search was conducted to identify economic evaluations of interventions in depression, launched on Medline and the Cost-Effectiveness Registry of the Tufts University using a ten-year time horizon (2008–2018). In a two-stepped screening process, cost-utility studies were selected by means of specified inclusion and exclusion criteria. Subsequently, relevant findings was extracted and, if not fully stated, calculated by the authors of this work.

**Results:**

Overall, 53 articles with 92 complete economic evaluations, reporting costs from healthcare payer/provider and societal perspective, were identified. More precisely, 22 estimations (24%) changed their results regarding the cost-effectiveness quadrant when the societal perspective was included. Furthermore, 5% of the ICURs resulted in cost-effectiveness regarding the chosen threshold (2% of them became dominant) when societal costs were included. However, another four estimations (4%) showed the opposite result: these interventions were no longer cost-effective after the inclusion of societal costs.

**Conclusions:**

Summarising the disparities in results and applied methods, the results show that societal costs might alter the conclusions in cost-utility analyses. Hence, the relevance of the perspectives chosen should be taken into account when carrying out an economic evaluation. This systematic review demonstrates that the results of economic evaluations can be affected by different methods available for estimating non-healthcare costs.

## Background

In 2015 depressive disorders affected 322 million people worldwide, making up more than 4.4% of the world’s population, and affecting women (5.1%) more than men (3.6%) [1]. Thus, even though different mental disorders can be highlighted, depression is considered as one of the most prominent mental disorders, as it is ranked fourth in the top ten causes with the largest amount of years lived with disability in Europe [2]. The disease not only causes high levels of distress and negative effects in those who suffer from it, even leading to early retirement or premature mortality, but also in their relatives, making it a growing public health issue worldwide [3, 4].

The prevalence of depression varies slightly by age with almost 10% of young adults and more than 11% of adolescents [5]. This results in several challenges concerning the individual productivity and work ability leading to a downward spiral of unemployment and financial burdens [6, 7]. To ease the symptoms of depression, many effective interventions and therapeutic approaches, such as cognitive therapy or medication, are available. However, especially antidepressants can cause various side effects [8]. Furthermore, due to the underestimation of the disease prevalence and possible concerns about the associated costs, the adverse effects of medication and stigmatization, many people suffering from depression remain without any treatment [9]. Besides this, depressive disorders are strongly associated with stigmatization. More precisely, evidence suggests that public stigma, defined as opinions about personal beliefs of what most people think, is (i) positively related to self-stigma and negatively associated with help-seeking for mental health problems [10], (ii) contributes to treatment discontinuity, and (iii) leads to poorer quality of life, self-esteem and worsened health status [11]. Consequently, the analysis of stigma effects and its economic consequences has led to an increasing interest in the existing literature [10], showing that stigma and discrimination related to mental health problems might lead to adverse economic effects, as it negatively affects employment, income and healthcare costs [12]. Moreover, anti-stigma campaigns for people with mental health problems have been proved to be a cost-effective alternative [13].

Taking into account all mentioned before, there is a growing public health interest in the economic effects of depression by a higher use of healthcare services (which range from 508€ to 24,069€) as well as vast labour productivity losses (ranging from 1963€ to 27,364€ per person per year) [14]. Across 28 European countries, the weight of societal costs can amount for more than 64% of the total economic burden of depression, with 76 billion euros in terms of losses related to premature mortality and morbidity [15]. The relevance of such costs has been confirmed in country-specific populations, where productivity losses represented half of the economic burden of depression [16].

Within this framework, economic evaluations are useful tools that can help decision-makers to prioritize healthcare interventions or policies in order to achieve not only improvements in health, but also ensuring the financial sustainability of public health systems. Particularly, the economic evaluations carried out in the field of depression have been performed in order to prevent depression [17], improve treatment adherence [16] or compare alternative treatments or interventions [18]. In spite of the existing amount of literature, comparability of economic evaluations poses several challenges [19, 20]. One of the main reasons is the selection of the most appropriate perspective [21]. Taking into account the importance of non-healthcare costs, it seems to be necessary to focus on encouraging their inclusion in any economic evaluation as well as emphasizing its role, as it has been done in other areas [22]. However, to the best of our knowledge, there is no evidence in the field of depression that has assessed the relevance of including or excluding costs beyond the healthcare ones. Therefore, the main aim of this paper is to analyse the role played by non-healthcare costs (labour productivity loss and/or informal care costs) in the economic evaluations carried out in any intervention for people with depression. More precisely, we would determine whether the inclusion/exclusion of societal costs could alter the results and conclusions of the economic evaluations in any intervention for such target population.

## Method

### Data source and search strategy

A systematic literature review was performed with the aim of identifying economic evaluations of any intervention in depression, taking into account the PRISMA methodology. It has not been prospectively registered anywhere. To identify economic evaluations of any intervention in depression, the search strategy was conducted in Medline using the following key words: “cost-benefit analysis” OR “quality adjusted life year” OR “cost-benefit” OR “economic evaluation” OR “cost-effectiveness” OR “cost-utility” OR “economic analysis” AND “depression” OR “depress*”, whereby MeSH terms and natural key words in titles and abstracts were combined. In order to ensure the sensitivity of the strategy, we searched for “depression” in the Cost-Effectiveness Analysis (CEA) Registry from the Tufts University. This publicly available comprehensive database uses a formalized review process to identify original economic evaluations containing cost-utility analysis (CUA) and to provide detailed information on these studies [23]. Both search strategies were limited to a period of 10 years from 30 November 2008 to 30 November 2018. The studies’ eligibility criteria included i) being an original study published in a peer-reviewed scientific journal ii) being an economic evaluation, more precisely a cost-utility analysis or a CEA and CUA, of any intervention related to depression regardless of whether the intervention was performed in patients with depression or to prevent depression; iii) in case of being an economic evaluation in more than one disease (i.e. anxiety), costs for depression were reported separately or it was explicitly stated that a majority of the participants were depressed; iv) including societal costs (informal care costs and/or productivity losses) in the analysis; v) using quality-adjusted life years (QALYs) in CUA vi); providing results separately for each perspective applied (healthcare and societal perspective); vii) and being written in English.

### Data extraction

After removing duplicates, an assessment considering the inclusion and exclusion criteria and data extraction was conducted by LP, BR, JD and LH. While three researchers were responsible for the first revision of titles and abstracts (LP, BR, IA), the full-text screening and data extraction was carried out by JD and LH, and double checked by LP and BR. Whenever there was a disagreement in screening process, the paper was reviewed by a third researcher (WG).

We extracted the following variables from each included study: authors, year of publication, perspective (society or healthcare payer/provider), country, type of intervention (prevention, screening/diagnostic, pharmaceutical therapy, non-pharmaceutical intervention, combined intervention, collaborative care), type of analysis (CUA or CEA/CUA), time horizon, discount rates used for costs and/or outcomes, study design, costs included, currency and type of sensitivity analysis (SA) (deterministic, probabilistic). Moreover, information about the analysis including the incremental costs, incremental QALYs, incremental cost utility ratio (ICUR), authors’ conclusions, whether the inclusion of societal costs changed the results or the conclusion about the adoption of the assessed intervention as well as the threshold were excerpted. In case of incomplete or misleading information, original authors were not contacted. To improve the comparability of the results, the incremental costs and ICURs were standardized by inflating the original currency to euros in 2018 prices using the Harmonised index of consumer prices [24]. Supplementary information can be obtained from the authors on request.

The underlying concepts of healthcare and societal costs followed Drummond et al. [25]. Therefore, healthcare costs encompass e.g. intervention costs, outpatient (incl. general practitioners and specialists) and inpatient services, medication and societal service costs. On the contrary, societal costs are defined as lost resources in consequence of absenteeism, presenteeism, premature death and costs of informal care [25]. We focus on the distinction between the healthcare payer/provider perspective and societal perspective. While the healthcare payer perspective includes the aforementioned health-care costs, the societal perspective further considers societal costs.

## Results

**Fig. 1:**
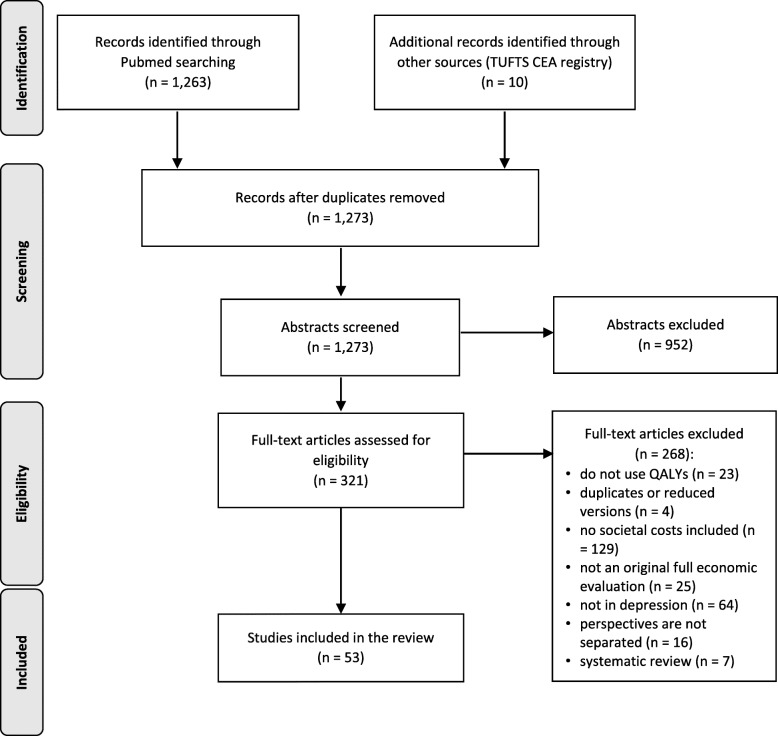
PRISMA flow diagram of the search strategy

By the initial search, 1273 articles were identified, of which 1263 were found in Medline and additional ten by the Tufts CEA registry. After reviewing all abstracts, 952 studies were excluded as duplicates or did not meet inclusion criteria. Three hundred twenty-one publications remained for the full text screening, of which 268 were excluded because they did not use QALYs (23 articles), four were identified as duplicated or reduced versions of an included publication, in 129 no societal costs were included and 25 did not include a complete economic evaluation or did not focus on depression (64 articles). Furthermore, we identified seven that followed a review design and 16 publications that did not report the perspectives of interest separately. Thus, a total of 53 articles met the full inclusion criteria and were therefore included in this review [26–78] (Fig. 1).

### Study characteristics

The majority of the 53 economic evaluations were carried out in the Netherlands (28%) [29, 30, 38, 41–43, 49, 51, 54, 57, 67, 71–73, 76] and the United Kingdom (26%) [28, 33, 35, 36, 44, 46, 47, 50, 52, 60–62, 75, 78]. Five studies derive from Spain [27, 39, 63, 64, 66] and four respectively from the United States [45, 48, 68, 69] and Germany [31, 32, 37, 40]. Three studies analysed data from Sweden [55, 56, 59] and another two from India [58, 77]. The remaining six studies used data from Japan [65], Greece [53], Canada [74], Belgium [26], Korea [34] and Finland [70].

Concerning the perspective, 22 of the studies (42%) applied the societal perspective [30, 38, 40–43, 45, 48, 49, 53–57, 59, 63, 66, 67, 69, 71–73, 76]. Eighteen studies conducted an evaluation considering both positions [26–29, 31, 32, 34, 37, 39, 50, 51, 58, 62, 64, 65, 74, 75, 77] and 12 focused on the healthcare perspective and calculated societal costs separately [33, 35, 36, 44, 46, 47, 52, 60, 61, 65, 70, 78]. One study did not explain which perspective was used [68]. Nevertheless, it was possible to extract costs and consequences separately for healthcare and society from the data included in the results table.

Considering the intervention type, 26% of the studies compared two or more different pharmaceuticals [26, 28, 34, 36, 38, 53, 55–57, 59, 62, 66, 69, 70], whereby the main part of the articles evaluated non-pharmaceutical interventions such as cognitive behavioural therapy or other psycho-educational therapies. Collaborative care interventions were evaluated in eight studies [27, 40, 42–44, 49, 60, 72] and seven articles focused on a combination of pharmaceutical and psychological interventions [46, 50, 54, 58, 65, 67, 75]. Preventive approaches [29, 32, 39] and screening or diagnostic tools [31, 45, 48] were used in three studies respectively. In almost all cases, standard care or treatment as usual was the comparator, regardless of the individual intervention type.

The majority (70%) of the 53 economic evaluation studies included CEA as well as CUA. Regarding the study design, most of the publications reported data from randomized controlled trials (RCT) [27–33, 35, 38–44, 46, 47, 49–52, 54, 58, 60–64, 66, 67, 69, 71–73, 76–78] and 16 studies used a modelling approach for the calculations [26, 34, 36, 37, 45, 48, 53, 55–57, 59, 65, 68, 70, 74, 75].

In 16 studies, both aspects of societal costs were included [29–33, 35, 44, 49–51, 58, 68, 71–73, 76]. The other studies used either productivity losses [26, 27, 34, 36–43, 45–48, 52–57, 59, 61, 63–67, 69, 70, 74, 75, 77, 78] or costs of informal care only [28, 60, 62]. The approach to estimate societal costs was not always explicitly depicted. When stated, the approach to value productivity losses was almost balanced between the human capital approach [31–33, 37, 39, 40, 46, 50, 61, 63, 64, 67, 70, 73, 75, 77, 78] and the friction costs method [26, 30, 38, 41–43, 49, 51, 52, 54, 57, 71, 72, 76], whereas one study used both [29]. In terms of the friction costs method, the replacement time varied between 123 and 161 days. Regarding informal care given by relatives or friends, most of the studies applied the proxy good method [28–33, 44, 49, 51, 60, 62, 71–73, 76] and only two used the opportunity cost method for their estimations [35, 50]. A comprehensive overview of the main characteristics relative to the methods and other context of cost estimation used is given in Table 1.

**Table 1:** Summary of the main characteristics of the selected studies

Authors. publication year	Country	Perspective	Intervention Type	Type of Economic evaluation	Time horizon	Discount rate	Studytype/ Type of model	Costs included	Currency (base year)	Type of sensitivity analysis
Annemans et al. 2014 [26]	BE	Healthcare and societal perspective	Pharmaceutical therapy	CUA	1 year	NA	Decision tree model	Direct costs: intervention costs, primary care Indirect costs: absenteeism, suicide	EUR (2011)	Deterministic and probabilistic
Aragones et al. 2014 [27]	ES	Healthcare and societal perspective	Collaborative care	CEA, CUA	1 year	NA	RCT	Direct costs: health care, intervention Indirect costs: absenteeism	EUR (2011)	Probabilistic
Banerjee et al. 2013 [28]	UK	Healthcare and societal perspective	Pharmaceutical therapy	CUA	13 weeks.39 weeks	NA	RCT	Direct costs: medication, health and social care Indirect costs: informal care	GBP (2009)	Deterministic
Biesheuvel-Leliefeld et al. 2018 [29]	NL	Healthcare and societal perspective	Prevention	CEA, CUA	1 year	NA	RCT (multi-center)	Direct costs: primary & secondary care, mental health care, home care, medication, intervention Indirect costs: absenteeism, presenteeism, informal care	EUR (2013)	Deterministic
Bosmans et al. 2008 [30]	NL	Societal perspective	Non-pharmaceutical intervention	CEA, CUA	1 year	NA	RCT	Direct costs: primary & secondary care, non-health care, medication Indirect costs: absenteeism, informal care	EUR (2002)	Probabilistic
Brett-schneider et al. 2017 [31]	DE	Healthcare and societal perspective	Screening/diagnostic	CUA	6 months	NA	RCT	Direct costs: inpatient & outpatient care, psychotherapist, medication, nursing care Indirect costs: absenteeism, informal care	EUR (2012)	Probabilistic
Buntrock et al. 2017 [32]	DE	Healthcare and societal perspective	Prevention	CEA, CUA	1 year	NA	RCT (pragmatic)	Direct costs: health care use, out-of-pocketIndirect costs: absenteeism, presenteeism, informal care	EUR (2013)	Deterministic and probabilistic
Chalder et al. 2012 [33]	UK	Healthcare perspective	Non-pharmaceutical intervention	CEA, CUA	1 year	3.5% (costs)	RCT (pragmatic)	Direct costs: primary & secondary care, intervention, medication, patient & carers Indirect costs: absenteeism	GBP (2009)	Probabilistic
Choi et al. 2016 [34]	KR	Healthcare and (limited) societal perspective	Pharmaceutical therapy	CUA	1 year	NA	Markov model	Direct costs: psychiatrist, AE, outpatient care, emergency,, laboratory tests, medication Indirect costs: absenteeism, suicide	KRW (2014)	Deterministic and probabilistic
Dixon et al. 2016 [35]	UK	Healthcare perspective	Non-pharmaceutical intervention	CUA	1 year	NA	RCT (multicentre)	Direct costs: primary & secondary care, medication, inpatient, ambulance, intervention, personal social services, out-of-pocket Indirect costs: absenteeism	GBP (2012/13)	Deterministic and probabilistic
Ekman et al. 2012 [36]	UK	Healthcare perspective	Pharmaceutical therapy	CEA, CUA	5 years	3.5% (costs and QALYs)	DES model	Direct costs: inpatient and outpatient, medication Indirect costs: absenteeism	GBP (2011)	Deterministic and probabilistic
Evans-Lacko et al. 2016 [37]	DE	Healthcare and societal (employer) perspective	Non-pharmaceutical intervention	CUA	27 months	3.5% (costs)	Decision tree model	Direct costs: screening, GP visits, primary care, inpatient, psychotherapist Indirect costs: absenteeism, presenteeism	EUR (2013)	Deterministic
Eveleigh et al. 2014 [38]	NL	Societal perspective	Pharmaceutical therapy	CUA	1 year	NA	RCT (cluster)	Direct costs: health services & resources, intervention Indirect costs: absenteeism	EUR (2012)	Probabilistic
Fernandez et al. 2018 [39]	ES	Healthcare and societal perspective	Prevention	CUA	1.5 years	3.5% (costs)	RCT (cluster)	Direct costs: medication, intervention Indirect costs: absenteeism, presenteeism	EUR (2012)	Deterministic and probabilistic
Gensichen et al. 2013 [40]	DE	Societal perspective	Collaborative care	CEA, CUA	2 years	NA	RCT (pragmatic)	Direct costs: psychiatric inpatient care, outpatient psychologic care, psychiatrists & GP visits, medication Indirect costs: absenteeism	EUR (2006)	Probabilistic
Gerhards et al. 2010 [41]	NL	Societal perspective	Deterministic and probabilistic	CEA, CUA	1 year	NA	RCT	Direct costs: healthcare use, costs for patient & family Indirect costs: absenteeism, presenteeism	EUR (2007)	Probabilistic
Goorden et al. 2015 [42]	NL	Societal perspective	Collaborative care	CUA	1 year	NA	RCT (cluster)	Direct costs: health care use Indirect costs: absenteeism, presenteeism	EUR (2013)	Probabilistic
Goorden et al. 2014 [43]	NL	Societal perspective	Collaborative care	CUA	13 months	NA	RCT	Direct costs: health care use Productivity costs: absenteeism, presenteeism	EUR (2009)	Probabilistic
Green et al. 2014 [44]	UK	Healthcare perspective	Collaborative care	CEA, CUA	1 year	NA	RCT	Direct costs: intervention, health & social care service, supervision, specialists Indirect costs: absenteeism, informal care	GBP (2011)	Deterministic and probabilistic
Groessl et al. 2018 [45]	US	Societal perspective	Screening/diagnostic	CEA, CUA	3 years	3% (costs)	Markov model	Direct costs: medical services, intervention Indirect costs: absenteeism	USD (2016)	Deterministic
Hollinghurst et al. 2014 [46]	UK	Healthcare perspective	Combined intervention	CUA	1 year	NA	RCT	Direct costs: health & social care services, intervention, out-of-pocket Indirect costs: absenteeism	GBP (2010)	Deterministic
Hollinghurst et al. 2010 [47]	UK	Healthcare perspective	Deterministic and probabilistic	CEA, CUA	8 months	NA	RCT	Direct costs: primary and community contacts, mental health-related secondary care, social services, out-of-pocket Indirect costs: absenteeism	GBP (2007)	Probabilistic
Hornberger et al. 2015 [48]	US	Societal perspective	Pharmaceutical therapy	CEA, CUA	patient’s lifetime	3% (costs)	Decision tree model	Direct costs: medication, inpatient & outpatient, psychotherapy, intervention Indirect costs: absenteeism	USD (2013)	Deterministic and probabilistic
Joling et al. 2013 [49]	NL	Societal perspective	Collaborative care	CEA, CUA	1 year	NA	RCT	Direct costs: ambulatory care, home care & other support, day hospital, inpatient Indirect costs: absenteeism, informal care	EUR (2009)	Deterministic and probabilistic
Kessler et al. 2018 [50]	UK	Healthcare and societal perspective	Combined intervention	CEA, CUA	1 year	NA	RCT	Direct costs: inpatient & outpatient care, private counselling, prescription charges, over-the-counter medication, complementary therapies, private home care Indirect costs: absenteeism	GBP (2016)	Probabilistic
Kolovos et al. 2016 [51]	NL	Healthcare and societal perspective	Non-pharmaceutical intervention	CEA, CUA	13 months	NA	RCT	Direct costs: health care & non-health care, Indirect costs: absenteeism, presenteeism, informal care	EUR (2013)	Deterministic and probabilistic
Kuyken et al. 2015 [52]	UK	Healthcare perspective	Non-pharmaceutical intervention	CEA, CUA	2 years	3.5% (costs and QALYs)	RCT	Direct costs: primary & secondary care, medication, social care & voluntary sector services, intervention, out-of-pocket Indirect costs: absenteeism, presenteeism	GBP (2011/12)	Probabilistic
Maniadakis et al. 2013 [53]	GR	Societal perspective	Pharmaceutical therapy	CEA, CUA	2 years	3.5% (costs)	Markov model	Direct costs: inpatient care, outpatient visits, medication, laboratory tests, AE Indirect costs: absenteeism	EUR (2012)	Deterministic and probabilistic
Meuldijk et al. 2015 [54]	NL	Societal perspective	Combined intervention	CEA, CUA	1 year	NA	RCT	Direct costs: primary care, non-healthcare use Indirect costs: presenteeism, absenteeism	EUR (2013)	NA
Nordstrom et al. 2012 [55]	SE	Societal perspective	Pharmaceutical therapy	CEA, CUA	6 months	NA	Decision tree model	Direct costs: hospitalization, ambulatory care, medication Indirect costs: absenteeism	EUR (2009)	Deterministic and probabilistic
Nordstrom et al. 2010 [56]	SE	Societal perspective	Pharmaceutical therapy	CEA, CUA	6 months	NA	Decision tree model	Direct costs: inpatient & outpatient care, medication Indirect costs: absenteeism	EUR (2009)	Deterministic and probabilistic
Nuijten et al. 2012 [57]	NL	Societal perspective	Pharmaceutical therapy	CUA	26 weeks	NA	Decision tree model	Direct costs: inpatient care, consultations, medication Indirect costs: absenteeism	EUR (2010)	Deterministic
Patel et al. 2017 [58]	IN	Healthcare and societal perspective	Combined intervention	CEA, CUA	3 months	NA	RCT	Direct costs: outpatient, inpatient, medication, intervention, laboratory tests Indirect costs: absenteeism, informal care	USD (2015)	Probabilistic
Ramsberg et al. 2012 [59]	SE	Societal perspective	Pharmaceutical therapy	CEA, CUA	1 year	NA	Decision tree model	Direct costs: medication, primary care, specialist care Indirect costs: absenteeism	EUR (2009)	Probabilistic
Richards et al. 2016 [60]	UK	Healthcare perspective	Collaborative care	CEA, CUA	1 year	NA	RCT (cluster)	Direct costs: primary, secondary & community care, social care, out-of-pocket Indirect costs: informal care	GBP (2011)	Deterministic and probabilistic
Richards et al. 2017 [61]	UK	Healthcare perspective	Non-pharmaceutical intervention	CEA, CUA	1.5 years	3.5% (costs and QALYs)	RCT	Direct costs: hospital services, primary care, social service, complementary services, medications Indirect costs: absenteeism	GBP (2013/14)	Deterministic and probabilistic
Romeo et al. 2013 [62]	UK	Healthcare and societal perspective	Pharmaceutical therapy	CEA, CUA	39 weeks	NA	RCT	Direct costs: inpatient, day hospital, outpatient, social care, occupational therapy, emergency Indirect costs: informal care	GBP (2009/10)	Deterministic
Romero-Sanchiz et al. 2017 [63]	ES	Societal perspective	Non-pharmaceutical intervention	CEA, CUA	1 year	NA	RCT	Direct costs: medications, medical tests, use of health-related services Indirect costs: absenteeism	EUR (2014)	Probabilistic
Rubio-Valera et al. 2013 [64]	ES	Healthcare and societal perspective	Non-pharmaceutical intervention	CEA, CUA	6 months	NA	RCT	Direct costs: publicy & privately funded primary & secondary care, tests, hospitalisation, medications Indirect costs: absenteeism	EUR (2009)	Deterministic
Sado et al. 2009 [65]	JP	Healthcare perspective	Combined intervention	CEA, CUA	1 year	NA	Decision tree model	Direct costs: medications, consultant & psychotherapy fees, inpatient Indirect costs: absenteeism	JPY (2005)	Deterministic and probabilistic
Serrano-Blanco et al. 2009 [66]	ES	Societal perspective	Pharmaceutical therapy	CUA	6 months	NA	RCT	Direct costs: medications, GP visits, specialized medical visits, emergency, inpatient Indirect costs: absenteeism	EUR (2001)	Deterministic
Simons et al. 2017 [67]	NL	Societal perspective	Combined intervention	CEA, CUA	32 weeks	NA	RCT	Direct costs: health care use, medications, intervention Indirect costs: absenteeism, presenteeism	EUR (2012)	Deterministic
Simpson et al. 2009 [68]	US	NA	Non-pharmaceutical intervention	CEA, CUA	1 year	NA	Decision tree and Markov model	Direct costs: inpatient, GP visits, emergency, medications Indirect costs: absenteeism, informal care	USD (2006)	Deterministic
Snedecor et al. 2010 [69]	US	Societal perspective	Pharmaceutical therapy		2 months/ 6 months	NA	RCT + modelling	Direct costs: medication, medical & health services Indirect costs: absenteeism, presenteeism	USD (2007)	Deterministic and probabilistic
Soini et al. 2017 [70]	FI	Healthcare perspective	Pharmaceutical therapy	CUA	1 year	NA	Decision tree and Markov model	Direct costs: psychiatrist & GP visits, psychotherapist, hospitalisations, medications Indirect costs: absenteeism	EUR (2014)	Deterministic and probabilistic
Stant et al. 2009 [71]	NL	Societal perspective	Non-pharmaceutical intervention	CEA, CUA	3 years	3 and 5% (costs)	RCT	Direct costs: inpatient & community care, healthcare, medications, intervention, non-medical Indirect costs: absenteeism, informal care	EUR (2003)	Probabilistic
van der Aa et al. 2017 [72]	NL	Societal perspective	Collaborative care	CEA, CUA	2 years	NA	RCT (multicenter)	Direct costs: primary & secondary care, medications, intervention Indirect costs: absenteeism, presenteeism, informal care	EUR (2013)	Deterministic and probabilistic
van Eeden et al. 2015 [73]	NL	Societal perspective	Non-pharmaceutical intervention	CEA, CUA	1 year	NA	RCT (multicenter)	Direct costs: care provider utilization, complementary care, home care, medications, intervention, patient & family Indirect costs: absenteeism, informal care	EUR (2012)	Deterministic
Vasiliadis et al. 2017 [74]	CA	Healthcare and societal perspective	Non-pharmaceutical intervention	CEA, CUA	40 years	NA	DES model	Direct costs: medical and non-medical Indirect costs: absenteeism	CAD (NA)	Deterministic
Vataire et al. 2014 [75]	UK	Healthcare and societal perspective	Combined intervention	CUA	5 years	3.5% (costs and QALYs)	DES model	Direct costs: inpatient, AE, GP & psychiatrist visits, medication, Indirect costs: absenteeism, suicide	GBP (2011)	Probabilistic
Warmerdam et al. 2010 [76]	NL	Societal perspective	Non-pharmaceutical intervention	CEA, CUA	12 weeks	NA	RCT	Direct costs: medical & non-medical, intervention, out of pocket, family & patients Indirect costs: absenteeism	EUR (2007)	Probabilistic
Weobong et al. 2017 [77]	IN	Healthcare and societal perspective	Non-pharmaceutical intervention	CUA	1 year	NA	RCT	Direct costs: healthcare use Indirect costs: absenteeism, informal care	USD (2015)	Probabilistic
Wiles et al. 2014 [78]	UK	Healthcare perspective	Non-pharmaceutical intervention	CEA, CUA	1 year	NA	RCT	Direct costs: healthcare use, intervention, social services Indirect costs: absenteeism	GPB (2010)	Probabilistic

The calculations were verified by SA in almost every study, except one publication [54]. Most of them used a probabilistic SA [27, 30, 31, 33, 38, 40–43, 47, 50, 52, 58, 59, 63, 71, 75–78] or a combination of both types (deterministic and probabilistic) of SA [26, 32, 34–36, 39, 44, 48, 49, 51, 53, 55, 56, 60, 61, 65, 69, 70, 72]. Thirteen articles evaluated a time horizon of less than 1 year [28, 31, 47, 55–58, 62, 64, 66, 67, 69, 76]. Twenty-five monitored the study population for 1 year [26, 27, 29, 30, 32–35, 38, 41, 42, 44, 46, 49, 50, 54, 59, 60, 63, 65, 68, 70, 73, 77, 78] and 15 evaluated a larger time horizon within a range from 13 months to lifetime [36, 37, 39, 40, 43, 45, 48, 51–53, 61, 71, 72, 74, 75]. In the latter cases, 11 of these analyses used a discount rate between 3 and 5% for discounting the costs [33, 36, 37, 39, 45, 48, 52, 53, 61, 71, 75]. However, only four of them discounted both costs and QALYs [36, 52, 61, 75].

### Results of economic evaluations

In 20 of the publications more than one result was calculated leading to a total of 92 individual results [26, 28, 38, 41, 44, 45, 49, 53, 56, 57, 59, 62, 63, 65, 67, 68, 70, 71, 74, 76]. All stated or obtained results were compiled and compared regarding the two perspectives by focussing on the changes in quadrants and conclusions (see Table 2).

**Table 2:** Summary of the economic results of the selected studies

Author & publication year	N° of estimation	Healthcare perspective				Societal perspective				Change in		threshold value
		∆Cost	∆QALY	ICUR (currency/QALY)	ICUR (2018€/QALY)	∆Cost	∆QALY	ICUR (currency/QALY)	ICUR (2018€/QALY)	results^**a**^	conclusions^**b**^	
Annemans et al. 2014 [26]	**1**	16	0.003	6352	6946	−258	0.003	−86,000	−94,049	**YES**	NO	30,000
	**2**	−5	0.004	− 1250	− 1367	− 387	0.004	−96,750	−105,805	NO	NO	
	**3**	−37	0.008	− 4625	− 5058	− 829	0.008	−103,625	−113,323	NO	NO	
	**4**	−50	0.009	− 5556	− 6076	− 902	0.009	−100,222	− 109,602	NO	NO	
	**5**	−104	0.016	− 6500	− 7108	−1618	0.016	−101,125	− 110,589	NO	NO	
	**6**	−128	0.015	− 8533	− 9332	− 1591	0.015	−106,067	− 115,994	NO	NO	
	**7**	− 118	0.006	−19,667	−21,507	− 843	0.006	− 140,500	−153,650	NO	NO	
Aragones et al. 2014 [27]	**8**	183	0.045	4056	4435	157	0.045	3499	3826	NO	NO	30,000
Banerjee et al. 2013 [28]	**9**	693	0.03	23,100	29,070	705	0.03	23,500	29,583	NO	NO	30,000
	**10**	404	0.05	8080	10,172	− 1106	0.05	−22,120	−27,962	**YES**	NO	
	**11**	− 289	0.02	−14,450	−18,191	− 1811	0.02	−90,550	−113,990	NO	NO	
Biesheuvel-Leliefeld et al. 2018 [29]	**12**	1107	0.03	33,025	35,216	2114	0.03	63,051	67,234	NO	NO	30,000
Bosmans et al. 2008 [30]	**13**	181	**−0.00045**	− 353,333	− 427,866	− 751	**−0.00045**	1,668,889	2,020,926	**YES**	NO	NA
Brettschneider et al. 2017 [31]	**14**	− 1031	0.0067	−153,881	−166,182	− 1309	0.0067	−195,373	− 210,991	NO	NO	50,000
Buntrock et al. 2017 [32]	**15**	135	0.01	13,500	14,396	134	0.01	13,400	14,289	NO	NO	20,000
Chalder et al. 2012 [33]	**16**	296	0.014	**20,834**	24,091	1813	0.014	**129,500**	149,744	NO	**YES**	30,000
Choi et al. 2016 [34]	**17**	−50,130	0.0131	− 3826,718	− 2765	−623,229	0.0131	−47,574,733	−34,379	NO	NO	NA
Dixon et al. 2016 [35]	**18**	177	0.031	5710	7463	324	0.031	10,452	13,662	NO	NO	30,000
Ekman et al. 2012 [36]	**19**	323	0.038	8591	11,076	485	0.043	11,173	14,405	NO	NO	30,000
Evans-Lacko et al. 2016 [37]	**20**	1280	0.05	25,600	27,298	2107	0.05	42,140	44,936	NO	NO	50,000
Eveleigh et al. 2014 [38]	**21**	−57	**−0.02**	2850	3039	− 1631	**−0.02**	70,180	75,790	NO	NO	80,000
	**22**	−97	**− 0.03**	3233	3448	1088	**− 0.03**	−36,267	−39,166	**YES**	NO	
Fernandez et al. 2018 [39]	**23**	24	0.02	1194	1289	−16	0.02	− 819	− 884	**YES**	NO	30,000
Gensichen et al. 2013 [40]	**24**	989	0.02	38,429	44,579	− 1322	0.02	−66,092	−76,668	**YES**	NO	NA
Gerhards et al. 2010 [41]	**25**	− 484	**−0.01**	48,400	55,508	− 1787	**− 0.01**	178,700	204,943	NO	YES^**c**^	80,000
	**26**	−83	**−0.01**	8300	9519	−451	**− 0.01**	45,100	51,723	NO	NO	
Goorden et al. 2015 [42]	**27**	1173	0.02	53,717	57,281	− 1131	0.02	−56,550	−60,301	**YES**	NO	80,000
Goorden et al. 2014 [43]	**28**	− 709	**−0.05**	14,589	16,346	− 2226	**− 0.05**	44,520	49,882	NO	NO	NA
Green et al. 2014 [44]	**29**	271	0.019	14,248	18,139	− 313	0.019	−16,465	−20,961	**YES**	NO	30,000
	**30**	316	0.051	6198	7875	− 1246	0.051	−24,434	−31,042	**YES**	NO	
Groessl et al. 2018 [45]	**31**	− 2918	0.10	−29,180	−27,459	− 2598	0.10	−25,980	−24,448	NO	NO	50,000
	**32**	− 6087	0.17	−35,806	−33,694	− 5810	0.17	−34,176	−32,161	NO	NO	
Hollinghurst et al. 2014 [46]	**33**	851	0.057	14,911	18,527	1039	0.057	18,228	22,649	NO	NO	30,000
Hollinghurst et al. 2010 [47]	**34**	−25	0.034	−17,173	− 29,240	− 330	0.034	− 9706	−16,526	NO	NO	30,000
Hornberger et al. 2015 [48]	**35**	− 3711	0.316	−11,744	− 9484	− 3764	0.316	−11,911	− 9619	NO	NO	50,000
Joling et al. 2013 [49]	**36**	75	0.04	**1875**	2101	4149	0.04	**157,534**	168,375	NO	**YES**	30,000
	**37**	187	0.006	31,167	34,920	2631	0.006	438,299	491,086	NO	NO	
Kessler et al. 2018 [50]	**38**	69	0.00935	**7380**	10,280	463	0.00935	**49,572**	69,052	NO	**YES**	20,000
Kolovos et al. 2016 [51]	**39**	−38	0.01	**− 3800**	− 4052	1579	0.01	**157,900**	168,375	**YES**	**YES**	30,000
Kuyken et al. 2015 [52]	**40**	124	**−0.04**	− 3103	− 4018	449	**−0.04**	−11,229	−14,618	NO	NO	NA
Maniadakis et al. 2013 [53]	**41**	350	0.026	13,682	14,776	14	0.026	547	613	NO	NO	50,000
	**42**	269	0.034	7959	8595	− 215	0.034	− 6324	− 7086	**YES**	NO	
	**43**	154	0.015	10,591	11,438	−28	0.015	− 1867	− 2092	**YES**	NO	
	**44**	273	0.03	9183	9917	−129	0.03	− 4300	− 4818	**YES**	NO	
Meuldijk et al. 2015 [54]	**45**	1880	0.005	376,000	400,943	4795	0.005	959,000	1,022,619	NO	NO	33,600
Nordstrom et al. 2012 [55]	**46**	−16	0.00865	− 1831	− 2052	− 169	0.00865	−19,555	−21,910	NO	NO	33,600
Nordstrom et al. 2010 [56]	**47**	−41	0.025	− 1647	− 1846	−507	0.025	−20,264	−22,704	NO	NO	33,600
	**48**	−17	0.025	− 687	− 770	− 483	0.025	−19,308	−21,633	NO	NO	
Nuijten et al. 2012 [57]	**49**	103	0.0062	16,363	18,116	−263	0.0062	−42,419	−46,963	**YES**	NO	80,000
	**50**	126	0.0166	7553	8362	− 1992	0.0166	−120,000	−132,854	**YES**	NO	
Patel et al. 2017 [58]	**51**	46	0.005	9333	7991	5	0.005	957	819	NO	NO	NA
Ramsberg et al. 2012 [59]	**52**	14	0.0036	3732	4181	−123	0.0036	−34,167	−38,282	**YES**	NO	NA
	**53**	−159	0.0045	−35,333	−39,589	− 327	0.0045	−72,667	−81,418	NO	NO	
	**54**	−11	0.0052	− 2115	− 2370	− 206	0.0052	−39,615	−44,387	NO	NO	
	**55**	−55	0.0072	− 7639	− 8559	− 325	0.0072	−45,139	−50,575	NO	NO	
	**56**	−79	0.0086	− 9186	−10,292	− 404	0.0086	−46,977	−52,634	NO	NO	
	**57**	− 147	0.0117	−12,564	−14,077	−588	0.0117	−50,256	− 56,309	NO	NO	
	**58**	− 179	0.0131	−13,664	−15,310	− 673	0.0131	−51,374	−57,561	NO	NO	
Richards et al. 2016 [60]	**59**	271	0.019	14,248	18,102	−313	0.019	−16,465	−20,918	**YES**	NO	20,000
Richards et al. 2017 [61]	**60**	− 343	0.05	− 6865	− 8661	− 2070	0.05	−41,392	−52,220	NO	NO	30,000
Romeo et al. 2013 [62]	**61**	693	0.03	23,100	28,702	705	0.03	23,500	28,842	NO	NO	30,000
	**62**	404	0.05	8080	10,040	−1106	0.05	−22,120	−27,149	**YES**	NO	
	**63**	−289	0.02	−14,450	−17,954	−1811	0.02	−90,550	− 111,135	NO	NO	
Romero-Sanchiz et al. 2017 [63]	**64**	−632	0.0567	−11,153	−11,743	− 644	0.0567	− 11,390	− 11,993	NO	NO	25,000
	**65**	− 466	0.0751	− 6204	− 6533	−479	0.0751	− 6381	− 6719	NO	NO	
	**66**	−322	0.0793	− 4060	− 4274	− 409	0.0793	− 5160	− 5434	NO	NO	
	**67**	− 295	0.0824	− 3576	− 3765	41	0.0824	497	523	**YES**	NO	
Rubio-Valera et al. 2013 [64]	**68**	962	0.01	3592	4025	1866	0.01	9872	11,061	NO	NO	30,000
Sado et al. 2009 [65]	**69**	27,411	0.08	**342,638**	2887	− 693,858	0.08	**−8,673,225**	−73,089	**YES**	**YES**	30,000
	**70**	27,411	0.03	**913,700**	7700	−693,858	0.03	**−23,128,600**	−194,904	**YES**	**YES**	
Serrano-Blanco et al. 2009 [66]	**71**	71	**−0.06**	− 1180	− 1444	639	**−0.06**	−10,643	−13,020	NO	NO	NA
Simons et al. 2017 [67]	**72**	423	0.07	6043	6526	1141	0.07	16,300	17,603	NO	NO	80,000
	**73**	1052	0.13	8092	8739	1741	0.13	13,392	14,463	NO		
Simpson et al. 2009 [68]	**74**	NA	NA	34,999	34,286	NA	NA	6667	6531	NO	NO	50,000
	**75**	NA	NA	− 1123	− 1100	NA	NA	− 7621	− 7466	NO	NO	
Snedecor et al. 2010 [69]	**76**	174	0.0058	30,000	26,060	81	0.0058	13,881	12,058	NO	NO	50,000
Soini et al. 2017 [70]	**77**	−223	0.0134	−16,642	−17,523	− 1074	0.0134	−80,149	−84,392	NO	NO	NA
	**78**	−128	0.0166	− 7711	− 8119	− 957	0.0166	−57,651	−60,702	NO	NO	
	**79**	−110	0.025	− 4400	− 4633	− 720	0.025	−28,800	−30,325	NO	NO	
	**80**	−238	0.0276	− 8623	− 9080	− 1390	0.0276	−50,362	− 53,028	NO	NO	
Stant et al. 2009 [71]	**81**	653	**−0.21**	− 3110	− 3727	1616	**−0.21**	− 7695	− 9222	NO	NO	NA
	**82**	1231	**−0.16**	− 7694	− 9220	1644	**−0.16**	−10,275	−12,314	NO	NO	
	**83**	713	**−0.04**	−17,825	−21,362	1054	**− 0.04**	−26,350	−31,579	NO	NO	
van der Aa et al. 2017 [72]	**84**	− 1154	0.03	−38,467	−41,019	− 877	0.03	−29,233	−31,172	NO	NO	30,000
van Eeden et al. 2015 [73]	**85**	1281	0.01	**107,455**	116,045	− 1913	0.01	**−160,390**	−173,211	**YES**	**YES**	40,000
Vasiliadis et al. 2017 [74]	**86**	− 1604	0.17	− 9435	− 7687	− 2590	0.17	−15,235	−12,412	NO	NO	NA
	**87**	−1604	0.17	−9435	−7687	− 1904	0.17	−11,200	− 9125	NO	NO	
Vataire et al. 2014 [75]	**88**	− 243	0.078	− 3115	− 5056	− 1400	0.078	−17,949	−29,128	NO	NO	30,000
Warmerdam et al. 2010 [76]	**89**	455	0.01	**45,500**	52,182	256	0.01	**22,609**	25,929	NO	**YES**	30,000
	**90**	405	0.01	**40,500**	46,448	147	0.01	**11,523**	13,215	NO	**YES**	30,000
Weobong et al. 2017 [77]	**91**	−18	0.011	− 1721	− 1474	− 155	0.011	−14,438	−12,362	NO	NO	16,060
Wiles et al. 2014 [78]	**92**	850	0.057	14,911	19,138	814	0.053	15,358	17,237	NO	NO	30,000

^a^ changes in results regarding the cost-effectiveness quadrant (e.g. from cost-effective to dominant/dominated)

^b^ change in conclusions regarding the chosen threshold

^c^ not included in the results because the interpretation of the ICUR regarding the chosen threshold is inappropriate in this quadrant (negative incremental costs and QALYs)

In seven studies, the economic evaluation of the intervention resulted in negative incremental QALYs [30, 38, 41, 43, 52, 65, 71]. Another three studies calculated incremental costs higher than the corresponding national willingness-to-pay threshold per QALY from both perspectives, thus resulting in the intervention not being cost-effective at all [29, 49, 54]. In one of these studies, only the unadjusted intention-to-treat analysis was not cost-effective, while adjusted analysis was cost-effective from the healthcare perspective [49]. Regarding the differences in incremental costs, 19 estimations from 14 studies showed cost savings when societal costs were included [26, 28, 38–40, 42, 44, 53, 57, 59, 60, 62, 65, 73]. However, only two of these results ended up in a decision change concerning the ICUR [65, 73]. From the 16 studies that explicitly conducted an evaluation from both perspectives, the majority of the studies did not identify a substantial change in the cost-effectiveness of the focused intervention(s) [26–29, 31, 32, 34, 37, 39, 58, 62, 74, 75, 77]. Nevertheless, 20 single results out of these studies led to lower incremental costs by inclusion of societal costs [26–28, 31, 32, 34, 39, 58, 62, 74, 75, 77]. Irrespective of the perspective of the evaluation, 15 single estimations accounted for increasing incremental costs when societal costs were included [28, 29, 33, 35–37, 45, 46, 50, 51, 54, 62–64, 67].

From the healthcare perspective, ten out of twelve papers reported that the focused intervention dominated the comparator [47, 61, 70] or had a positive ICUR [33, 35, 36, 44, 46, 60, 78] below the threshold applied. When the societal costs were included, the majority of them showed no relevant changes to the direct costs-results [35, 36, 46, 47, 52, 61, 70, 78]. In two cases, the results changed from the intervention having a positive ICUR below the used threshold to being dominant [44, 60]. One study demonstrated a conclusion change for both single results from not being cost-effective when adopting the healthcare perspective to highly dominant when societal costs were included [65]. However, one study changed from a positive ICUR to a value markedly above the chosen threshold of 30,000 British pounds with respect to the obtained data from the healthcare perspective [33].

In addition, when the societal perspective was selected, two results changed and became cost-effective compared to the analysis only including direct healthcare costs [73, 76]. One of these studies ascertained a complete change from a value high above the willingness-to-pay for one QALY to cost savings when societal costs were included, thus becoming not only cost-effective but also dominant [73]. In that special case the scientists evaluated an augmented cognitive behavioural therapy compared to a computerized cognitive training program in post-stroke depressive patients. Another eight economic estimations out of six studies changed from being below the threshold to dominate the standard care or other comparators [40, 42, 53, 57, 59, 73]. One of the interventions altered from being cost-effective to rise above the threshold when societal costs were included [49]. Further, one study changed in a similar way but results from negative incremental costs and negative incremental QALYs [41]. In most of the single results from the societal perspective, no important changes in terms of being cost-effective were obtained [45, 48, 53, 55, 56, 62, 67, 69, 72].

**Fig. 2:**
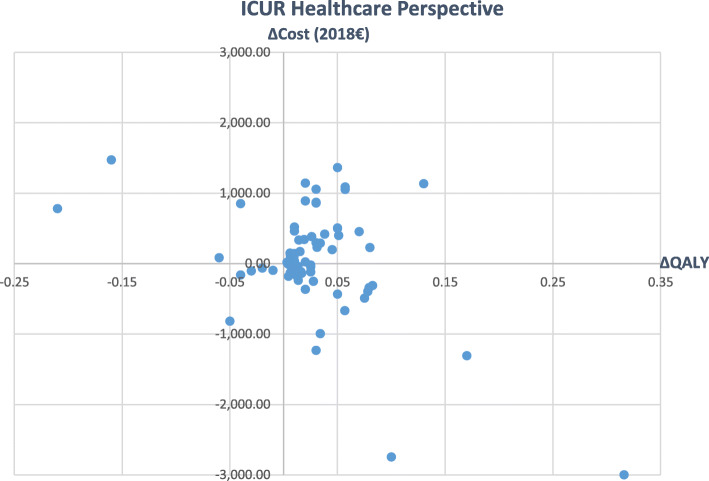
Incremental Cost-Utility Ratios from healthcare perspective

**Fig. 3:**
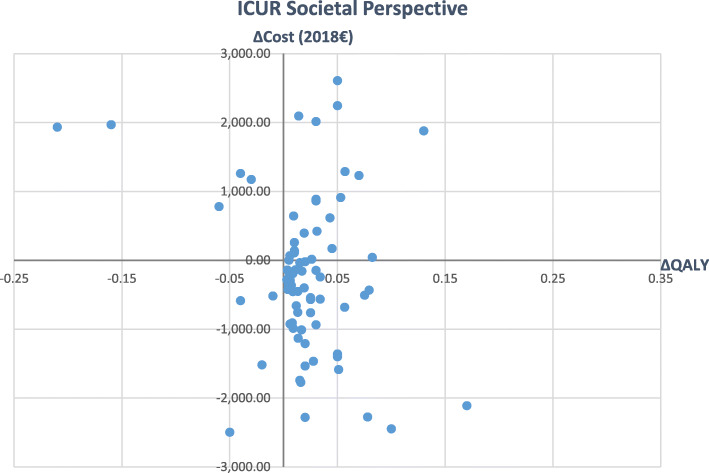
Incremental Cost-Utility Ratios from societal perspective

Figures 2 and 3 give an overview of main variations in the inflated ICURs from healthcare to societal perspective (Please note, that outliers must be excluded for the visualization.). It can be seen that the inclusion of societal costs led to a wider spread of single values in all directions (see Figs. 2 and 3).

## Discussion

To the best of our knowledge, this is the first review that examines CUA-studies depending on the change in results by including or excluding societal costs (productivity losses and/or informal care costs) in the field of depression, which might even lead to changes in conclusions. Concretely, our results suggest that when societal costs were considered, some of the economic evaluations carried out in depression changed their conclusions/recommendations, as well as their results. More precisely, of the 92 economic evaluations coming from the 53 articles identified, 22 estimations changed their results regarding the cost-effectiveness quadrant when a societal perspective was included, while 9 estimations changed their conclusions in the decision-making regarding the chosen threshold. In fact, five economic evaluations became cost-effective (three of these single results became dominant) when societal costs were included, compared to the analysis which only included healthcare costs. However, in another four estimations the opposite result was found: these interventions were no longer cost-effective after the inclusion of societal costs. Furthermore, twelve economic evaluations changed from being below the threshold to dominate the standard of care or other comparators when the societal perspective was taken into account.

However, it should be noted that the estimations which calculated negative QALYs were limitedly included in the appraisal of the relevance of societal costs in depression. In fact, economic discussions about the appropriateness of the interpretation of incremental cost-utility or cost-effectiveness results are growing increasingly [79–81]. Especially in the case of negative health effects and lower costs, the ratio is positive as well as when observing positive incremental costs and QALYs. Then, results of Incremental Cost-Effectiveness Ratios in quadrant one and three are both positive but with a very different meaning. Considering the included article of Gerhards et al. the reported ICUR might lead to false conclusions without taking into account the underlying differences in costs and QALYs [41]. Alternative approaches like the net-monetary benefit analysis can help decision makers to overcome these pitfalls [82]. Otherwise the results would lead to the conclusion that including societal costs change results in an opposite way [83]. Even though the inclusion of societal costs changed the recommendations derived from the economic evaluations only in a low number of cases, the importance of revealing potential savings in terms of costs that affect not only the healthcare system but also the society as a whole should be considered.

Nevertheless, even though there are no previous studies that have performed such analysis in the field of depression, and therefore, no direct comparison could be done, the results obtained are in line with those in previous papers in which the authors aimed to analyse the role played by societal costs in economic evaluations in different therapeutic areas [22, 84–86]. Particularly, the consideration of productivity losses could alter the decisions regarding reimbursement of expensive drugs in almost one-third of the cases [85]. In fact, it seems that, depending on the patient’s profile, the type of societal costs included (productivity losses and/or informal care costs) might vary. In the case of depression, due to the profile of such populations where the mean age of onset ranges from 24 to 35 years of age, and where productivity losses might have a higher weight within the economic impact of such a disease [74, 75], it is more common to include only productivity losses in CUAs than taking into account informal care or both types of societal costs [87]. Thus, almost 94% of the economic evaluations in mental and behavioural disorders that include the societal perspective solely considered productivity losses as societal costs only, while informal care costs were only taken into account in 29% of those evaluations with a societal point of view. Moreover, it was striking that productivity losses were mostly based on absenteeism and less often on presenteeism or both aspects, even though there is sufficient evidence of the existence of presenteeism in depressive disorders [76, 77]. Actually, it has been estimated that presenteeism costs are five to ten times higher than productivity losses due to absenteeism among people with depression, with differences across age groups, educational level and countries being observed [88]. In addition, the relevance of the different approaches to estimate productivity losses could be doubtful, especially in case of narrower time frames. Almost three-quarters of the articles used a time horizon of 1 year or less whilst differences in absenteeism, depending on the approach to be used, are hard to see during such short periods of time. A 5-year horizon analysis showed, in fact, that absenteeism costs were largely increasing after those 5 years, with additional worse health outcomes among absenteeism reporters than presenteeism ones [89].

Regarding the role of informal caregivers, previous studies proved that the impact of informal care costs differed between studies, depending mainly on the disease considered [22, 84, 86]. These papers evidences the fact that informal care costs were only present in one of the economic evaluations considered, making visible that the role played by non-professional care costs in economic evaluations of depression are not quite frequent. However, there is a study which demonstrated that informal care costs could be quite relevant in the field of depression [46]. More precisely, this study, which changed from being cost-effective to rise above the threshold by including societal costs, had the aim of implementing a preventive intervention for caregivers of dementia patients to minimize their risk of developing a depressive disorder. The CUA of these family meetings includes QALYs as well as direct and indirect costs of caregivers and patients. Since the differences in QALYs were very small and informal care costs represented by far the largest contributor to total costs, it is not surprising that the societal perspective led to different results. Therefore, it should be taken into account that interventions for depression could also affect the family caregivers’ health and, in this case, costs and QALYs of caregivers should be considered in economic evaluations. In this sense, the societal perspective demands to incorporate not only societal costs but also effects on the health of caregivers, as well as other spill-over effects [90–94].

Another relevant aspect that should be highlighted is that costs due to (attempted) suicide were merely included in three out of the 53 studies, although it is known that the risk for suicide in depressed patients is much higher than in the general population [78]. The risk of suicide is closely related to social stigmatization of persons with depression, which remained fully unconsidered in the identified literature of this review [9, 79–82]. Therefore, due to the importance of such factor in populations with depression and its economic impact on this disease, further economic evaluations should include these cost components so as not to underestimate the real economic consequences of depression. For this purpose, economic evaluations should consider broader time horizons than the ones which are commonly used in this field.

CUAs of interventions for people suffering from depressive disorders include not only the relevant costs but also the estimated QALYs. Existing literature remarks challenges for using the QALY approach in the field of mental health. Although generic instruments seem to be able to reflect the impact of common conditions such as mild to moderate depression, there are general concerns regarding the measurement of Health-related quality of life (HRQoL) in different groups of patients [95]. There is a perceived need for improved instruments that measure health-related quality of life so that QALYs appropriately reflect the pain, suffering, and limitations experienced by people with mental illness [96]. This would help to better capture the effects of the interventions being evaluated. Hence, the reported incremental QALYs may fail to capture the interventions effect and therefore lead to inaccurate ICURs. In addition to the aspects mentioned above, different degrees of severity of depressive disorders can affect the results of economic evaluations. Thus, the societal perspective may be more relevant in case of severe depression, because of potentially higher costs due to presenteeism and absenteeism [97, 98]. As the extent of the disease is not always reported by the authors of the underlying studies and the current review focusses especially on the methodological issues of the involvement of societal costs in economic evaluations, this factor was not included in the analysis.

A few limitations of this review should be mentioned. First, several studies showed inconsistencies between the results described in text and tables. Moreover, some analyses were not fully consistent with the methods. In this case, it was not possible to include every single result stated in the selected studies. Due to the heterogeneity in the SA, we only took into consideration the results reported in the main analysis, leaving out the figures reported in the SA. Secondly, the methods applied in the studies varied widely in terms of time horizon and measurement of costs and QALYs. Some limitations refer to limited time resources. Although the initial search resulted in a large number of 1273 studies, it could have been reasonable to expand the timeframe and to extend the literature search to another database. However, as previous studies show, Tufts CEA registry ensures a more accurate search [99]. In this case, 1263 articles were found from PubMed and 1273 from Tufts, getting 10 additional articles from this registry. Limited time resources even restricted capabilities to contact the authors in case of incomplete or misleading information as well as the implementation of an additional quality assessment (e.g. the Consensus on Health Economic Criteria (CHEC)-list [100]). Therefore, the high heterogeneity and variability of the methods applied in the different economic evaluations might be considered when interpreting the results obtained.

## Conclusion

This study contributes to the existing literature by analysing whether the perspective (healthcare payer/provider or societal) of a CUA in the field of depression alters the results and conclusions of the evaluation. Our findings suggest that in some of the studies the inclusion of societal costs of depression leads to substantial changes in both results and conclusions, although wide methodological variations have also been observed. Thus, several analyses led to different conclusions when the intervention was evaluated from a societal compared to a healthcare payer perspective. The results revealed potential savings as well as increases from the evaluated interventions when such costs were included. However, and in purpose to improve comparability, economic evaluations should ideally consider the healthcare as well as the societal perspective leading to more appropriate recommendations. Additionally, future research should consistently follow established guidelines (e.g. CHEERS statement [101]) by reporting all relevant cost components as well as the methods of measurement. In brief, an issue that this paper highlights, is the need for considering the societal perspective when conceptualizing economic evaluations, especially among populations with depression where productivity losses could represent an important weight of its economic impact. Therefore, not considering such effect might lead to an inefficient allocation of resources when designing policies in such target populations.

## Data Availability

The data analysed during the current study are available from the corresponding authors on request.

## Abbreviations

AE: Adverse events
CEA: Cost-Effectiveness Analysis
CUA: Cost-Utility Analysis
GP: General practitioner
ICUR: Incremental Cost-Utility Ratio
QALY: Quality-adjusted life year
RCT: Randomized controlled trial
SA: Sensitivity analysis
